# Assessing Black Carbon and Iron Oxide Aerosols: A Comparative Study between Urban and Rural Environments in the Southeastern U.S.

**DOI:** 10.1021/acsestair.5c00400

**Published:** 2026-05-22

**Authors:** Shreya Suri, Lifei Yin, Bin Bai, Yuhan Yang, Dongli Wang, Andrew R. Metcalf, Nathan Chellman, Robert F. Swarthout, James Sherman, Pengfei Liu

**Affiliations:** † School of Earth and Atmospheric Sciences, 1372 Georgia Institute of Technology, Atlanta, Georgia 30332, United States; ‡ Department of Environmental Engineering and Earth Sciences, 2545 Clemson University, Clemson, South Carolina 29634, United States; § Division of Hydrologic Sciences, 34148 Desert Research Institute, Reno, Nevada 89512, United States; ∥ Department of Chemistry and Fermentation Sciences, 1801 Appalachian State University, Boone, North Carolina 28608, United States; ⊥ Department of Physics and Astronomy, Appalachian State University, Boone, North Carolina 28608, United States

**Keywords:** carbonaceous aerosols, iron-containing aerosols, PM_1_, single-particle soot photometer, light absorption

## Abstract

Black carbon (BC) and iron oxide (FeO*_x_*) aerosols represent important contributors to shortwave atmospheric heating and have been associated with adverse human health outcomes. Accurate measurement of BC and FeO*_x_* is crucial for understanding their roles in climate forcing and air quality. However, observational data—particularly for anthropogenic FeO*_x_* —remain limited. In this study, we use a modified Single Particle Soot Photometer to measure atmospheric concentrations of refractory BC (rBC) and FeO*_x_* particles in urban (Atlanta, GA) and rural (Boone, NC) environments in the southeastern U.S. We identify likely emission sources and estimate light absorption properties to assess the relative contributions of rBC and FeO*_x_* to atmospheric heating. Our findings indicate that rBC and FeO*_x_* concentrations and absorption are substantially higher in the urban environment, reflecting significant anthropogenic contributions to pollutant emissions. Assuming external mixing, we estimate that FeO*_x_* contributes 1.3% of total absorption by rBC at the urban site and approximately 0.6% at the rural site. These results highlight the spatial variability of light-absorbing aerosol components and the importance of localized assessments to better understand their impacts on air quality and climate.

## Graphical abstract

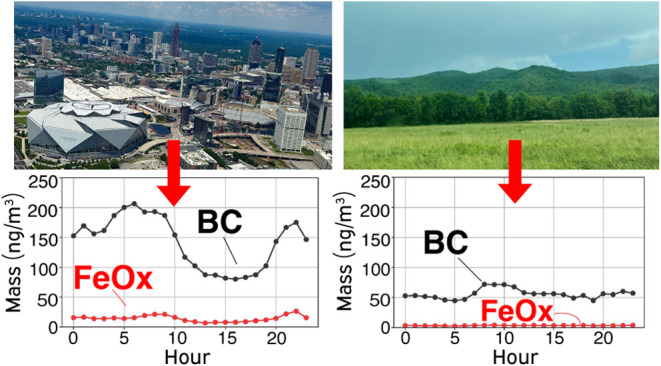

## 1 Introduction

Light-absorbing aerosols, such as black carbon (BC), light-absorbing organic carbon (i.e., brown carbon, BrC), and iron oxides (FeO*_x_*), play a key role in Earth’s climate system by absorbing shortwave radiation, reducing snow albedo, and directly heating the atmosphere and Earth’s surface.^1–3^ These aerosols are also associated with adverse public health outcomes, including respiratory, cardiovascular, and neurological effects.^4–7^

Due to its strong light absorption efficiency, relatively long atmospheric lifetime compared to other light-absorbing species, and substantial contribution to both climate warming and air quality degradation,^8^ BC is widely recognized as the most important light-absorbing aerosol, and is therefore the most intensively studied. BC is produced primarily by the incomplete combustion of fossil fuels, biofuels, and biomass.^9^ Traditional methods used to estimate BC include thermal-optical analysis, in which “organic carbon” (OC) is differentiated from “elemental carbon” (EC) through thermal volatilization and subsequent measurement of carbonaceous material.^10^ EC measurements—often loosely associated with the terms “soot” and “black carbon”— provide a direct assessment of nonvolatile carbonaceous mass, but can be influenced by charring artifacts in the presence of OC.^11,12^ BC can also be quantified via light absorption measurements, using instruments such as the photoacoustic spectrometer,^13^ aethalometer,^14^ and multiangle absorption photometer.^15^ These optical methods enable real-time, online measurements of BC (which is, by definition, optically defined), but require an assumed mass absorption coefficient (MAC) to convert absorption to BC mass, introducing potential bias.^16^ Additionally, absorption by dark brown carbon can be substantial even at long visible wavelengths,^17^ potentially leading to misclassification errors in optical-based BC measurements.

In contrast to BC, the concentrations, size distributions, and contributions of FeO*_x_* to light absorption remain poorly understood. FeO*_x_* particles originate from both natural and anthropogenic sources, such as mineral dust,^18–20^ engine exhaust and brake wear of motor vehicles,^21–23^ blast furnaces of iron manufacturing facilities,^24,25^ and coal and heavy oil combustion.^26^ Traditional methods measuring Fe-containing and other metallic aerosols have utilized filter-based measurements and source apportionment methods,^2,27,28^ which lack the temporal resolution and individual particle specificity needed for detailed characterization.

The Single Particle Soot Photometer (SP2; Droplet Measurement Technologies, Boulder, CO) offers a significant advancement in measuring the mass of refractory particles, including refractory BC (rBC) and FeO*_x_* particles, providing real-time, *in situ* measurements and detailed characterization of individual particles. The SP2 employs laser-induced incandescence to detect both the mass and number concentration of refractory particles.^29–32^ A modified version of the instrument incorporates an additional sensor to quantify the blue-to-red color ratio of incandescent emissions, enabling the differentiation between rBC and FeO*_x_* particles based on their distinct boiling points.^33^ Although the SP2 efficiently detects pure magnetite and hematite particles within its detection range, it has low efficiency in detecting natural mineral dust particles with mixed morphology and composition.^2,33,34^ Thus, FeO*_x_* particles detected by SP2 are most likely of anthropogenic origin,^2^ even though natural mineral dust remains the dominant global source of total atmospheric iron (Fe).^19,20,35–37^ This capability has only been applied in a limited number of field studies to date^2,27,28,34^ and, to our knowledge, has not yet been used to report FeO*_x_* measurements in the continental U.S.

Using this modified SP2, recent measurements in Asian cities^2,27,28^ and remote oceans^34^ identified FeO*_x_* particles associated with anthropogenic sources as an overlooked source of light-absorbing aerosols. Aircraft measurements over East Asia indicate that shortwave absorption by anthropogenic FeO_*x*_ aerosols contributes 4–7% of that attributed to rBC, making their radiative impact comparable to that of brown carbon (BrC) over the region.^2,27,28,38^ Moreover, a global modeling study incorporating these observations of FeO_*x*_ from continental Asian outflow along with ground-based observations from European outflow suggests that anthropogenic emissions of Fe-containing aerosols from combustion could be underestimated by up to 5-fold globally.^39^ In remote environments, measurements from various aircraft campaigns indicate that shortwave atmospheric heating from anthropogenic FeO_*x*_ is approximately 0.3 to 26% of that attributed to BC.^34^

The contribution of anthropogenic sources to FeO_*x*_ emissions in the continental U.S., however, remains largely uncertain. Offline electron microscopy measurements have shown that anthropogenic FeO_*x*_ particles, typically in the form of aggregated nanoparticles, are ubiquitous in urban atmospheres^2,4,40,41^ and roadside environments,^23^ with the primary FeO*_x_* phases identified in these aggregated nanoparticles being magnetite (Fe_3_O_4_),^2,4,21–23,40^ maghemite (γ-Fe_2_O_3_),^21,23^ and hematite (α-Fe_2_O_3_).^21,23,40^

In this study, we quantified the number and mass concentrations and characterized the size distributions of submicrometer rBC and anthropogenic FeO_*x*_ particles in two contrasting locations in the southeastern U.S., which is a region characterized by relatively elevated PM_2.5_ (particulate matter with aerodynamic diameter ≤ 2.5 μm) levels compared to other parts of the United States.^7,42–44^ Measurements were conducted in Atlanta, Georgia (urban) and Boone, North Carolina (rural, high-elevation site) using a modified SP2. We further identified likely major sources by analyzing the diurnal variations and characteristic size distributions of rBC and FeO_*x*_ particles. Based on the measurement results, we estimated rBC and FeO_*x*_ light absorption using a Mie-scattering optical model to assess their potential contribution to atmospheric heating. These observations provide insight into rBC and FeO_*x*_ variability across contrasting urban and rural environments in the southeastern U.S., contributing to a broader understanding of how light-absorbing aerosols impact air quality and climate challenges across different environments.

## 2 Methods

### 2.1 Study Sites

Field campaigns were carried out at two distinct sites representing urban and rural environments with different elevations. The locations of these sites are depicted in Figure S1, while meteorological data including temperature, precipitation, and relative humidity (RH) are presented in Figure S2.

### 2.2 Urban Site (ATL)

The urban ambient monitoring station was located on the rooftop of the Ford Environmental Science and Technology building on the Georgia Tech campus in Midtown Atlanta, Georgia (33.78°N, 84.40°W, 311 m a.s.l.) (Figure S1e). The aerosol inlet of the rooftop lab was located approximately 25 m above ground level. This site is situated in a densely populated area characterized by heavy vehicular traffic (approximately 800 m from interstate freeways I-75/85) and industrial activities. Recent studies^7,45^ using high-resolution air quality data sets have identified the Atlanta area as a regional hotspot for rBC, and have linked rBC exposure to significant health impacts, including incident dementia.^7^ The field campaign at the ATL site was conducted from August 9 to September 15, 2023. Meteorological data for this site were retrieved from the WeatherSTEM database associated with Georgia Tech’s Bobby Dodd Stadium weather station approximately 500 m away from the rooftop lab.^46^

### 2.3 Rural High-Elevation Site (APP)

The rural ambient monitoring station was located at the Appalachian Atmospheric Interdisciplinary Research (AppalAIR) facility on the campus of Appalachian State University in Boone, Watauga County, North Carolina (33.21°N, 81.69°W, 1076 m a.s.l.) (Figure S1d). The aerosol inlet was located at the top of a ∼34 m tower above the forest canopy. This high-elevation site, near the crest of the southern Appalachian Mountains, is exposed to air masses from all directions. It is characterized by low population (∼20,000 population in Boone, NC^47^), minimal industrial activity, and extensive forested areas. Even so, Boone experiences local traffic emissions from the university during weekdays and tourism during weekends and during the summer months. Additionally, motor vehicles registered in Watauga County are exempt from emission tests, further contributing to the potential local emissions from traffic.^48^ The field campaign at the APP site was conducted from June 22 to July 28, 2024. Meteorological data for this site were retrieved from the WeatherSTEM database associated with Appalachian State University’s Kidd Brewer Stadium weather station approximately 600 m away from the AppalAIR facility.^49^

### 2.4 Modified Single-Particle Soot Photometer (SP2)

The SP2 measures time-dependent scattering and incandescence signals from individual aerosol particles passing through an intracavity Nd:YAG laser beam operating at λ = 1064 nm.^29^ Refractory particles are brought to incandescence when they are heated by the laser beam to their boiling points (∼4000 K for rBC, ∼3000 K for FeO_*x*_).^29–32^ The induced incandescence signal is proportional to the amount of refractory material. The signals are detected by four optical detectors at two different gains for each detector, which enhance the size range of detection. The mass of individual particles is indirectly quantified by measuring the intensity of thermal emission (i.e., peak amplitude of incandescence signal) of the particle at its boiling point. Compared to the standard commercial version, the modified SP2 utilizes a narrow band photomultiplier tube (PMT) with increased sensitivity to red light detection (λ = 600–750 nm) as well as an additional incandescence detector—the blue band detector— that filters for shorter wavelengths in the visible spectrum (λ = 300–450 nm).^30^

### 2.5 Calibration of the SP2

The SP2 was calibrated using Aquadag (Acheson Industries Inc., Port Huron, MI, #9054), magnetite (Sigma-Aldrich, 310069), and hematite (Sigma-Aldrich, 310050) across different mass ranges. Aquadag is a commercial colloidal suspension of graphitic carbon in water that is often used as a calibration material for rBC due to its stable effective density and reproducible SP2 calibration curves within the same batch.^50,51^ In our study, the modified SP2 was calibrated with Aquadag over a mass range of 0.07 to 94 fg (corresponding to a nm mass-equivalent diameter (*D_m_*) range of approximately 46.3 to 511 nm for rBC assuming spherical morphology and an effective density of 1.8 g/cm^3^), magnetite over a range of 2.7 to 5950 fg (*D_m_* equivalent of 100 to 1300 nm assuming spherical morphology and an effective density of 5.17 g/cm^3^), and hematite over a range of 2.7 to 21900 fg (*D_m_* equivalent of 100 to 2000 nm assuming spherical morphology and an effective density of 5.24 g/cm^3^). Aquadag particles were aerosolized using an APEX-2Q nebulizer (Elemental Scientific Inc., Omaha, NE), while magnetite and hematite aerosols were generated using a small volume medical-grade nebulizer (Allied B&F Medical, #61400) capable of generating larger particles. Particles were selected using a wide-range Differential Mobility Analyzer (DMA 3083; TSI Inc., Shoreview, MN). A correction factor of 0.75 was applied to the calculated mass of individual rBC particles to account for the SP2′s higher sensitivity to Aquadag compared to ambient rBC particles.^51,52^ Calibration curves used for analysis are shown in Figure S3.

### 2.6 Classification of rBC and FeO_*x*_ Particles and Uncertainty Analysis

The different wavelength bands of the incandescence channels in the modified SP2 configuration allow for the discrimination between rBC and FeO_*x*_ particles.^33^ The ratio of the signal from the shorter wavelength band (λ = 300–450 nm, blue band) to the signal from the longer wavelength band (λ = 600–750 nm, red band)—known as the color ratio—serves as an indicator of boiling temperature, while the peak amplitude of the blue band incandescence signal serves as an indicator of the size of incandescing particles.^2,33^ Following the methodology utilized by Moteki et al.^2^ and Yoshida et al.,^27^ Figure S4 illustrates how rBC and FeO_*x*_ particles were distinguished based on the amplitude of their blue band low gain incandescence signals and their color ratios. Because the color ratio reflects intrinsic differences between rBC and FeO_*x*_ particles, plotting the color ratio against the blue band incandescence signal yields two distinct clusters corresponding to each particle type.^2,27,33^ The boundary line of the separation algorithm discriminating FeO_*x*_ from rBC (also shown in Figure S4) is therefore defined empirically as a separator between these two well-resolved clusters, rather than a strict physical threshold.^2^ This classification was validated by overlaying calibration data, which fall within their expected regions for rBC and FeO_*x*_ (Figure S4).

Size ranges defining the instrument detection limits for rBC and FeO_*x*_ particles were estimated from instrument responses using calibration data from both field campaigns. For the ATL campaign, the modified SP2 used in this study measured rBC particles within a range of approximately 42 nm ≤ *D_m_* ≤ 511 nm and could detect magnetite particles within the size domains of 250 nm ≤ *D_m_* ≤ 1200 nm. For the APP campaign, the SP2 detected rBC particles within a range of 54 nm ≤ *D_m_* ≤ 486 nm and magnetite particles within a range of 250 nm ≤ *D_m_* ≤ 1200 nm. Hematite particles were detected within the size range of 250 nm ≤ *D_m_* ≤ 1600 nm.

Final mass loading quantification utilized ATL Aquadag, ATL magnetite, APP postcampaign Aquadag, and APP postcampaign magnetite calibrations (Figure S3), assuming all detected ambient FeO_*x*_ particles were magnetite. All calibration data were employed to evaluate measurement uncertainty associated with variations in the calibration curves and the assumed FeO_*x*_ type.

### 2.7 Data Processing and Analysis

SP2 data was processed using a modified version^53^ of a Python package developed by the Atmospheric Radiation Management (ARM) user facility of the U.S. Department of Energy (DOE).^54^ This package converts raw SP2 data into number and mass concentrations and size distributions, replicating the functionality of the original IGOR-based software developed by DMT,^55^ while offering a more efficient, Python-based alternative optimized for data processing. Additional data analysis was also performed using Python.

Because the SP2 detects only a limited size range of rBC and FeO_*x*_ particles, we extended the size coverage by fitting the measured size distributions with log-normal functions. The fitted number- and mass-diameter distributions, *n̂*_*j*_(θ) and *m̂*_*j*_(θ), are expressed as

1 $${\mathrm{n̂}}_{j}\left(\theta \right)=\sum_{i=1}^{k}=\frac{N_{i}}{\sqrt{2\pi }\log_{10}\sigma_{g,i}\ln\left(10\right)}\exp\left[-\frac{{\left(\log_{10}D_{j}-\log_{10}D_{g}{,}_{i}\right)}^{2}}{2\left(\log_{10}\sigma g,i\right)}\right]$$

2 $${\mathrm{m̂}}_{j}\left(\theta \right)=\frac{\pi }{6}\rho D_{j}^{3}\sum_{i=1}^{k}\frac{N_{i}}{\sqrt{2\pi }\log_{10}\sigma_{g,i}\ln\left(10\right)}\exp\left[\frac{-{\left(\log_{10}D_{j}-\log_{10}D_{g,i}\right)}^{2}}{2\left(\log_{10}\sigma_{g,i}\right)}\right]$$

where *D*_*j*_ is the midpoint diameter of bin *j*, and θ = {*N*_*i*_, *D*_g,*i*_,σ_g,*i*_} _*i* = 1_^*k*^ is the set of fitted parameters describing a *k*-mode log-normal distribution. In this set, *N*_*i*_ is the number concentration, *D*_g,*i*_ is the geometric mean diameter, and σ_g,*i*_ is the geometric standard deviation of mode *i*. The material densities (ρ) are fixed at 1.8 g/cm^3^ for rBC and 5.17 g/cm^3^ for FeO_*x*_. The number of modes *k* is determined adaptively: if a single mode does not adequately reproduce both number and mass distributions, a second mode is added; if two modes remain insufficient, a third mode is used. No more than three modes are fitted.

To constrain both number and mass size distributions simultaneously, we minimized the following objective function

3 $$J\left(\theta \right)=\sum_{j=1}^{J}{\left[\log_{10}n_{j}^{\mathrm{obs}}-\log_{10}{\mathrm{n̂}}_{j}\left(\theta \right)\right]}^{2}+w\sum_{j=1}^{J}{\left[\log_{10}m_{j}^{\mathrm{obs}}-\log_{10}{\mathrm{m̂}}_{j}\left(\theta \right)\right]}^{2}$$

where $n_{j}^{\mathrm{obs}}=\frac{\mathrm{dN}}{d\ln D}$ and $m_{j}^{\mathrm{obs}}=\frac{\mathrm{dN}}{d\ln D}$ are the measured number- and mass-diameter distributions, respectively, and *w* is a scalar weight factor that balances number and mass residuals. The parameters θ are derived by minimizing the objective function *J*. This fitting algorithm ensures that a single set of parameters can reproduce both number and mass distributions consistently.

### 2.8 Light Absorption Calculations

Based on measured and fitted rBC and FeO_*x*_ size distributions, the absorption coefficients, *b*_abs_(λ) (Mm^–1^), for rBC and FeO_*x*_ particles were calculated using a Mie computational package (MATLAB Functions for Mie Scattering and Absorption, version 2)^56^ at λ = 500 nm assuming spherical particles. This wavelength was selected as a reference because it lies near the center of the visible spectrum and falls within the 500–550 nm range commonly used to report aerosol optical properties.^9,57^ As a first-order estimate, an external mixing state was assumed, with refractive indices of 1.94 + 0.66*i* ^2,58^ for rBC and 2.5 + 0.65*i* ^2,59^ for FeO_*x*_, under the assumption that all FeO_*x*_ particles were magnetite. As a sensitivity test, the *b*_abs_(λ) for FeO_*x*_ was also calculated assuming all particles were hematite, using a refractive index of 3.34 + 0.23*i* for λ = 500 nm.^60^

## 3 Results

### 3.1 rBC and FeO_*x*_ Correlations, Timeseries, and Diurnal Profiles

Figure 1 shows a timeseries of rBC and FeO_*x*_ mass concentrations along with wind speed and direction data for both ATL and APP sites. A timeseries of rBC and FeO_*x*_ number concentrations along with other meteorological variables (temperature, precipitation, RH) is presented in Figure S2. rBC concentrations are substantially higher at the ATL site than at the APP site, with campaign average number (58.4 #/cm^3^) and mass (150 ng/m^3^) concentrations approximately 2 and 2.5 times greater than those at APP (33.1 #/cm^3^, 58.2 ng/m^3^), respectively (Table 1). The contrast is even more pronounced for FeO_*x*_, with number (0.0570 #/cm^3^) and mass (15.1 ng/m^3^) concentrations at ATL roughly 6.5 and 4 times higher than those at APP (0.00882 #/cm^3^, 3.53 ng/m^3^; Table 1). Consequently, FeO_*x*_/rBC number and mass ratios are higher for ATL (Figure S2e,f,g,h). The higher rBC and FeO_*x*_ concentrations at the ATL site likely result from greater human activity in Atlanta, including denser traffic and industrial sources, as well as less obstructed transport pathways compared to Boone. In Boone, local surface emissions must navigate the denser forest canopy and hilly terrain, which may reduce measured concentrations.

**Figure 1:**
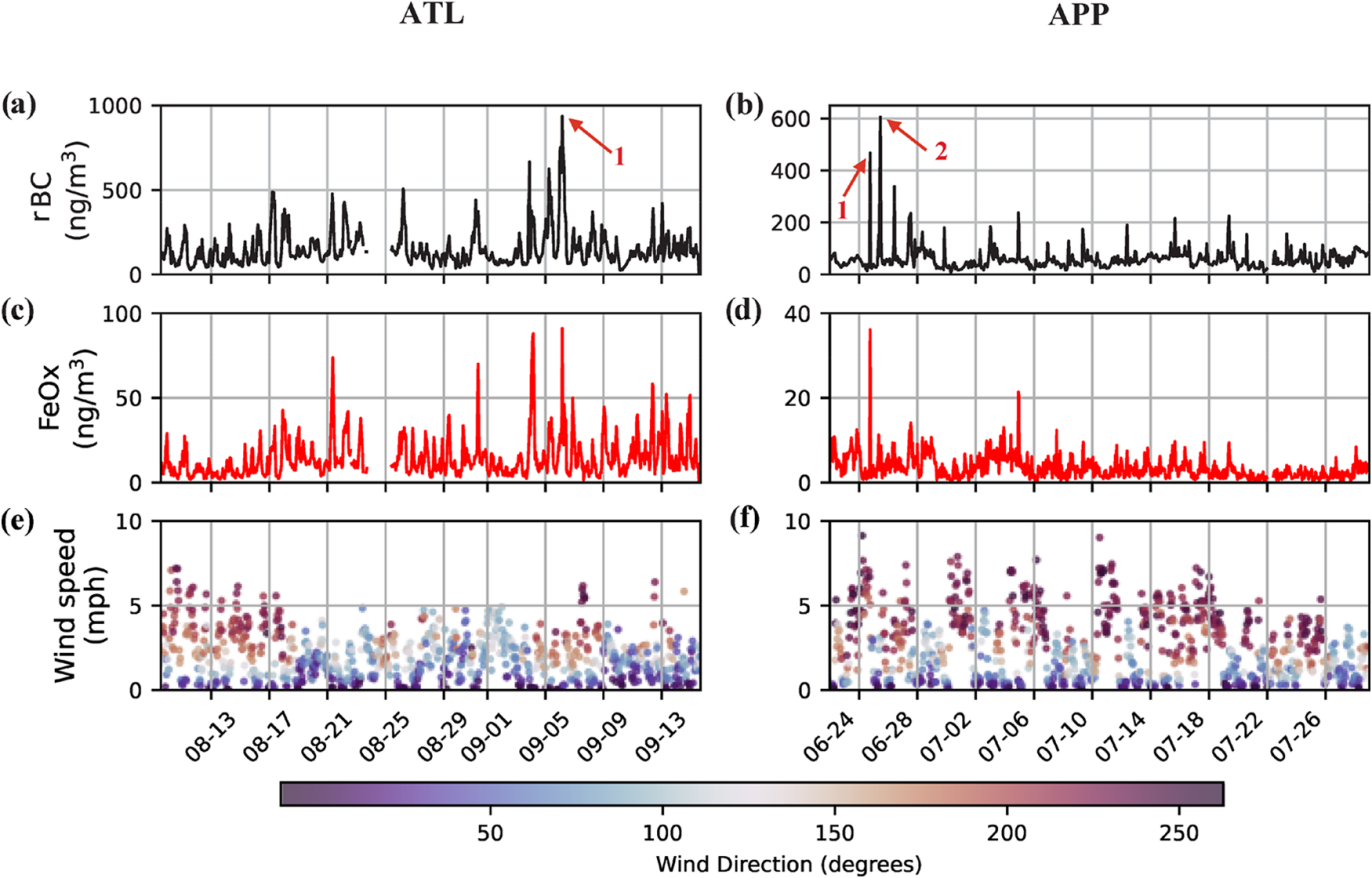
Timeseries of rBC and FeO_*x*_ mass concentration and wind speed/direction at Atlanta, GA (ATL; August 9–September 15, 2023) and Boone, NC (APP; June 22–July 28, 2024) sites. (a, b) rBC mass concentration, with red arrows (labeled ″1″ in panel a and “1” and “2” in panel b) indicating maximum rBC peaks corresponding to the highest rBC concentrations observed at each site. (c, d) FeO_*x*_ mass concentration. (e, f) Wind speed and direction. Wind data were obtained from WeatherSTEM sites at Georgia Tech’s Bobby Dodd Stadium (ATL) and Appalachian State University’s Kidd Brewer Stadium (APP).

**Table 1:** Summary of Measured and Fitted Total Number Concentration (*N*_tot_ *)*, Total Mass Concentration (*M*_tot_), and Total Population Absorption Coefficient (*b*_abs_) for ATL and APP Sites^a^

	ATL		APP	
	rBC	FeO_*x*_	rBC	FeO_*x*_
average *N*_tot_ (#/cm^3^)	58.4 (32.8, 69.1)	0.0570(0.0250, 0.0658)	33.1 (21.2, 37.6)	0.00882 (0.00518, 0.0110)
fitted *N*_tot_ (#/cm^3^)	60.2	0.0613	37.2	0.0165
average *M*_tot_ (ng/m^3^)	150 (85.4, 180.5)	15.1 (6.91, 17.4)	58.3 (37.0, 68.4)	3.53 (1.62, 4.51)
fitted *M*_tot_ (ng/m^3^)	157	18.7	58.8	7.39
measured total *b*_abs_ (Mm^–1^)	0.902	0.0113	0.367	0.00184
fitted total *b*_abs_ (Mm^–1^)	0.940	0.0121	0.382	0.00247

a Fitted values were derived from lognormally fitted size distributions (lognormal fitting is shown in Figure 5). Values in parentheses represent 25 and 75% quartiles, respectively, calculated from hourly data

Figure 2 illustrates the diurnal profiles of rBC and FeO_*x*_ concentrations at both sites for weekdays and weekends. A clear “weekday-weekend effect”—referring to observable differences in emission patterns between weekdays and weekends driven primarily by changes in anthropogenic activity^61,62^ — is evident during morning rush hours (between 7 and 10 am) for both rBC (Figure 2a,2c) and FeO_*x*_ (Figure 2e,g) at ATL, and for rBC at APP (Figure 2b,d). The pattern for FeO_*x*_ at APP is less distinct (Figure 2f,h). These results suggest that local traffic emissions are a significant cosource of rBC and FeO_*x*_ at ATL, contributing to their synchronized morning peaks. Furthermore, the distinct weekday-weekend effect observed for rBC at APP indicates that local anthropogenic emissions still play a significant role in the rural setting.

**Figure 2:**
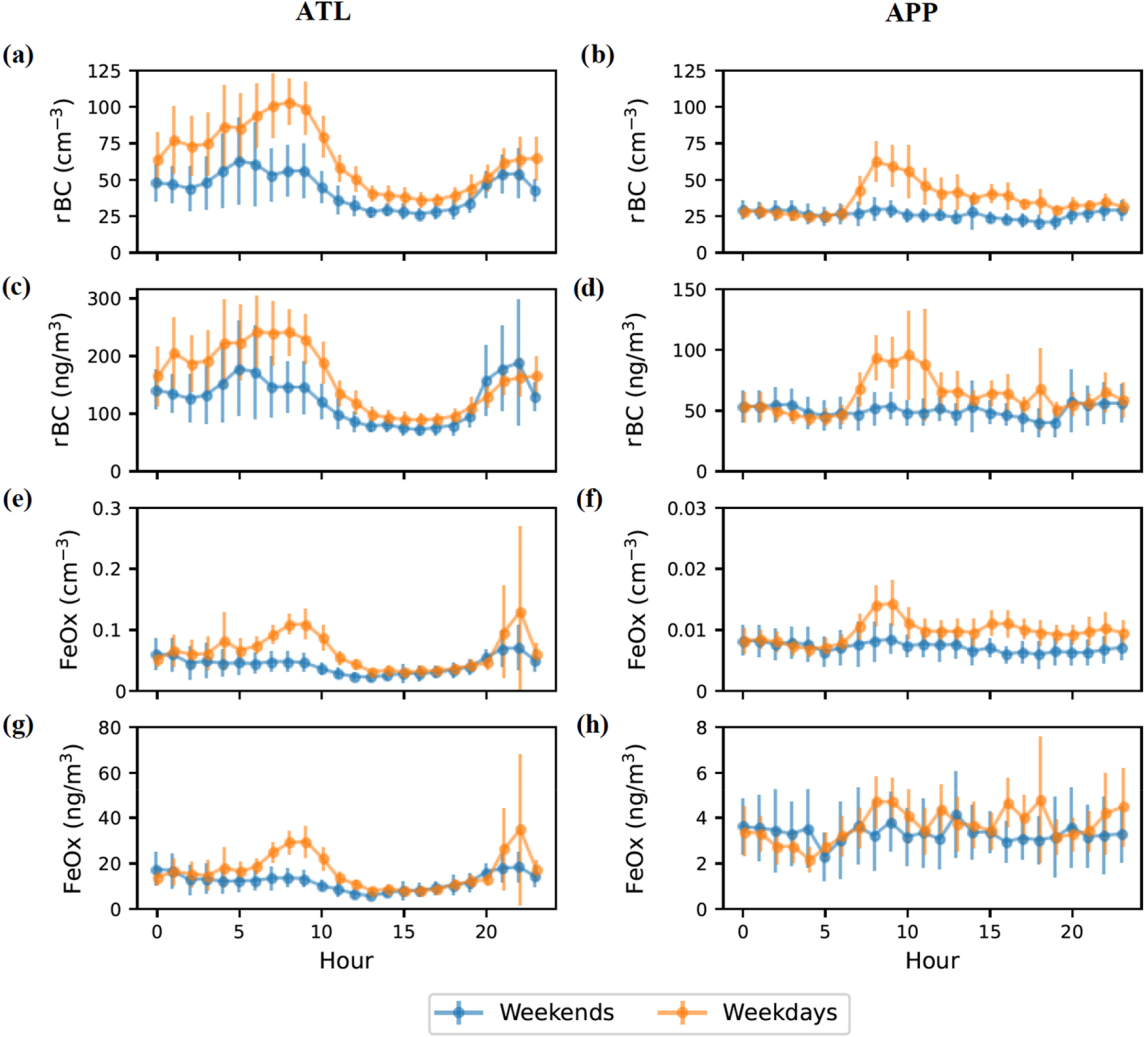
Hourly averages for (a, b) rBC number concentration, (c, d) rBC mass concentration, (e, f) FeO_*x*_ number concentration, and (g, h) FeO_*x*_ mass concentration for weekdays (orange line) and weekends (blue line) at the ATL and APP sites. Error bars indicate ±2 × standard error (s.e.), corresponding to 95% confidence intervals of the mean.

Figure 3 shows the correlation between rBC and FeO_*x*_ concentrations, with positive correlations observed at both sites. However, the *R* ^2^ values for both number and mass concentrations are notably greater at the ATL site compared to the APP site, reinforcing the interpretation that rBC and FeO_*x*_ exhibit stronger covariation at ATL and supporting the hypothesis that traffic emissions serve as a dominant shared source in urban environments. In contrast, the weaker correlations at APP and the more distinct separation of rBC and FeO_*x*_ mass concentrations suggest that these pollutants at APP are influenced by a broader range of sources, such as long-range transport, regional pollution, and natural emissions. In addition to emissions, the diurnal variations in boundary layer height may also contribute to the observed covariation of the two pollutants.

**Figure 3:**
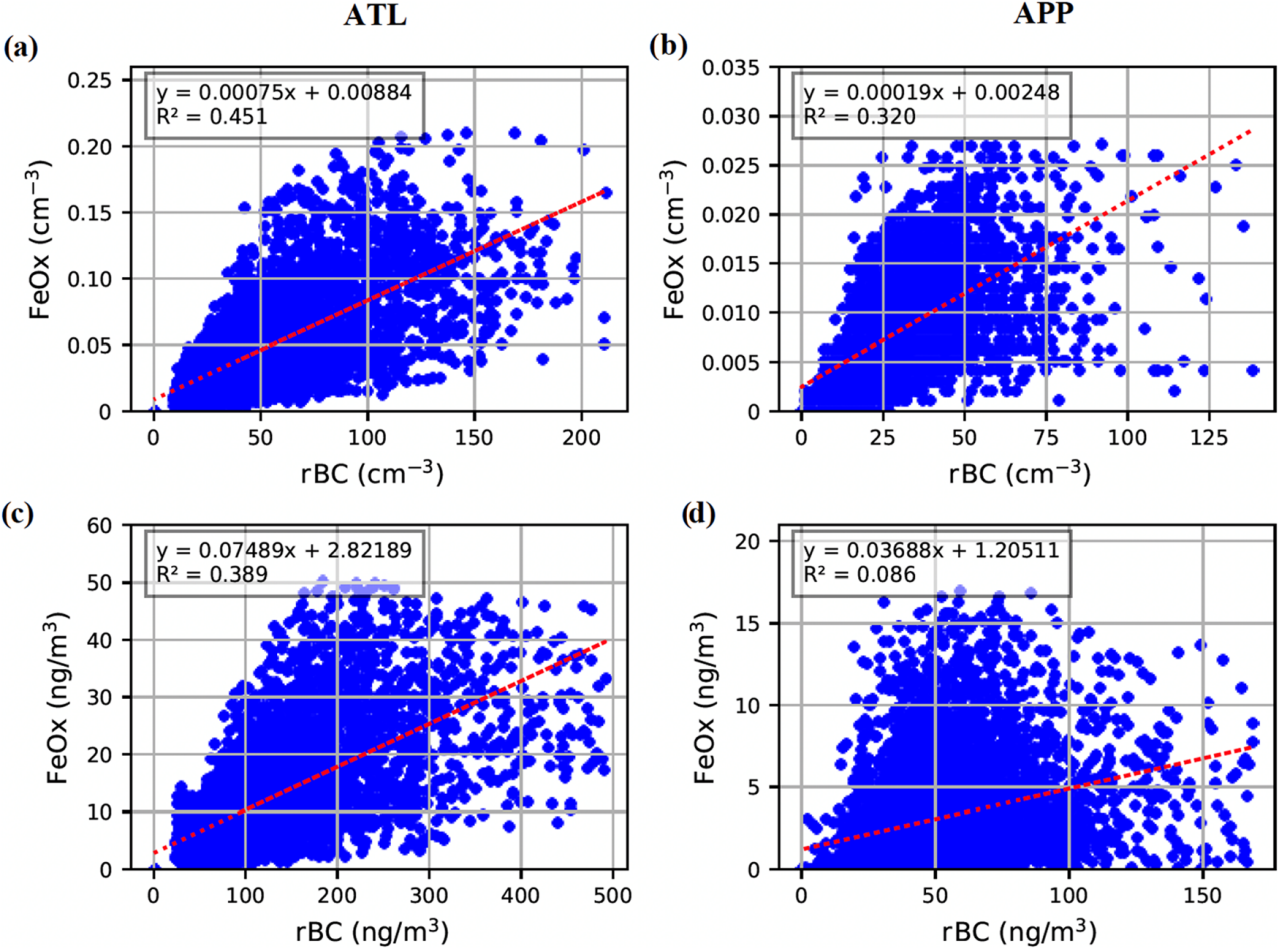
Correlation between FeO_*x*_ and rBC concentrations. FeO_*x*_ vs rBC number concentration for (a) ATL and (b) APP sites, and FeO_*x*_ vs rBC mass concentration for (c) ATL and (d) APP sites. All regressions are statistically significant (*p* < 0.001).

### 3.2 Relationship between Pollutant Concentrations and Wind Speed/Direction

Figure 4 further examines the relationship between pollutant mass concentrations and wind speed/direction presented in Figure 1 (Figure S5 provides additional context on the relationship between pollutant number concentrations and wind speed/direction). At the ATL site, both rBC and FeO_*x*_ exhibited maximum concentrations during periods of low wind speed (e.g., maximum ATL rBC peak 1 labeled "1" in Figure 1a) and decreased with increasing wind speed (Figure 4a,4c). In contrast, the relationship between pollutant concentrations and wind speed at the APP site is less apparent (Figure 4b,d). Notably, however, certain peaks in rBC concentrations at the APP site, labeled "1" and "2" in Figure 1b, coincide with higher wind speeds and appear as outlier scatter points in Figure 4b,d. These events were also captured by an aethalometer deployed at the APP site (Figure S6a). Due to the unusually high rBC mass concentrations and high wind speeds, we hypothesize that these peaks represent biomass burning episodes, which we investigate further in the subsequent section. Our observations suggest that local emissions are the primary sources of rBC and FeO_*x*_ in ATL, while episodic pollutant transport is more important at APP for the studied period. Across both sites, the strongest winds originate from the southwest, while the weakest winds come from the north and east directions (see Figure S7 for pollutant wind roses). These wind patterns are likely influenced by the Bermuda High, a persistent high-pressure system that dominates the southeastern U.S. during the summer months. This high-pressure system is known to increase the strength of southwest winds while attenuating those from the north and east.^63^

**Figure 4:**
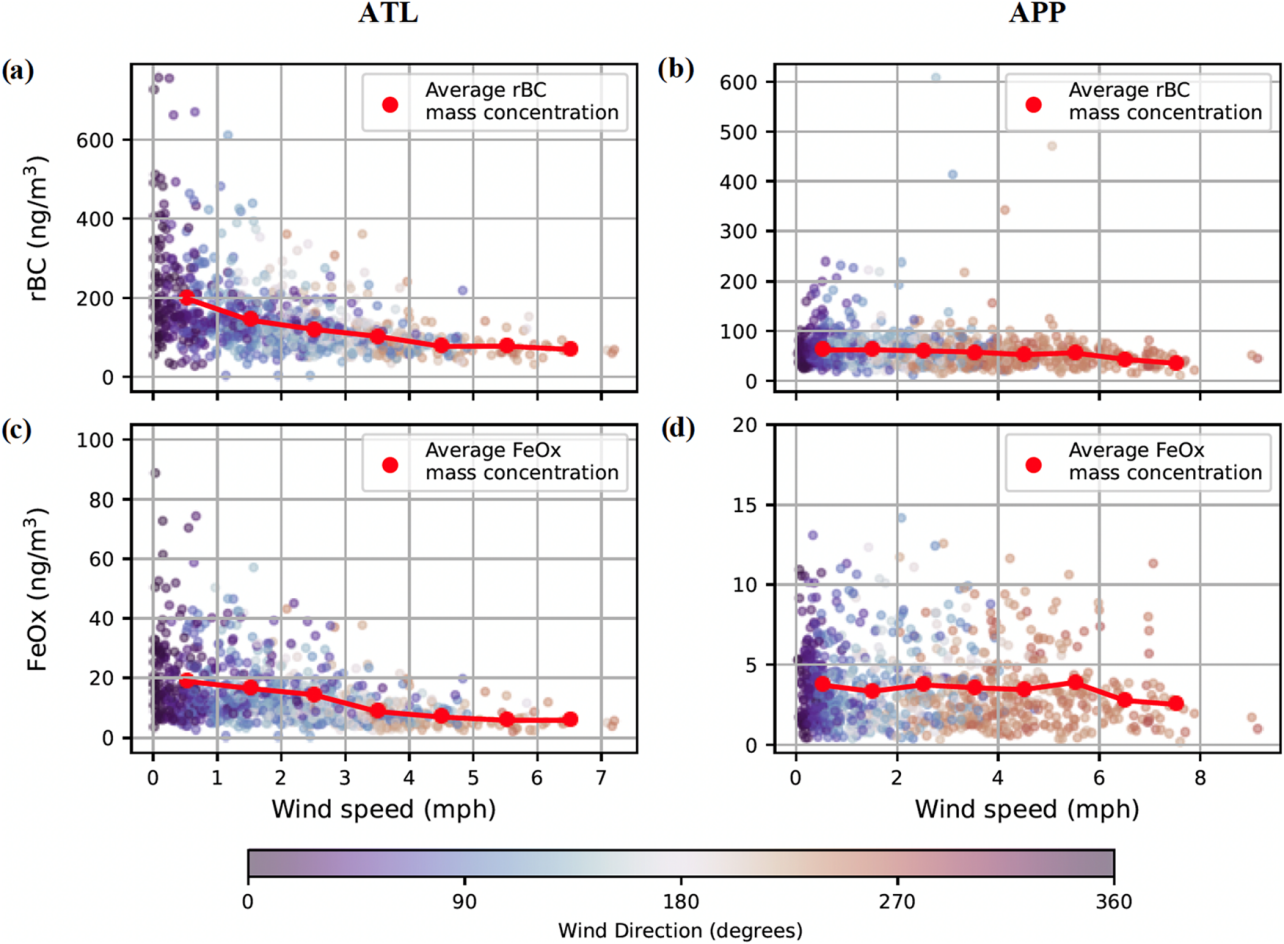
Relationship between pollutant mass concentration, wind speed, and wind direction. Scatterplots of (a, b) rBC and (c, d) FeO_*x*_ mass concentration plotted against wind speed for ATL and APP sites. Colored markers represent wind direction, while larger red markers represent mean pollutant mass concentration for each bin.

### 3.3 rBC and FeO_*x*_ Size Distributions and Relative Absorption

Table 2 presents the mode diameters and geometric standard deviations (σ_g_) of rBC number and mass distributions for various ATL and APP data sets, including campaign averages, morning rush hour periods, and identified peaks (i.e., ATL peak 1, labeled “1” in Figure 1a, and APP rBC peak 1 and peak 2, labeled ″1″ and ″2″ in Figure 1b). rBC size distributions for all ATL data sets exhibit similar mode diameters and σ_g_ values, with only one mode identified in each case based on our lognormal fitting algorithm. Notably, the size distributions for ATL rBC peak 1 are nearly identical to those of ATL campaign average (refer to Figure S8 for normalized size distributions; ATL rBC peak 1 is not shown due to this similarity). This consistency across ATL data sets— particularly the similarity between the ATL campaign average and ATL morning rush hour period—suggests that traffic emissions are the dominant source of rBC in Atlanta.

**Table 2:** Summary of Lognormal Fitting Parameters (Derived from eqs 1 and 2) for rBC Number and Mass Distributions of Various Data Sets, Including ATL Campaign Average, APP Campaign Average, ATL Morning Rush Hour, APP Morning Rush Hour, ATL rBC Peak 1, APP rBC Peak 1, and APP rBC Peak 2^a^

	number of modes	*N*_*i*_ (cm^–3^)	*D*_g_ (nm)	σ_g_
ATL average	1	59.7	106	1.53
ATL rush hour	1	100.1	102	1.53
ATL peak 1	1	189	106	1.54
APP average	2	10.0, 25.5	90, 120	1.35, 1.50
APP rush hour	3	45.8, 8.38, 5.19	86, 137, 140	1.30, 1.48, 1.20
APP peak 1	2	187, 53.0	107, 180	1.44, 1.40
APP peak 2	3	57.1, 38.6, 4.83	94, 181, 298	1.41, 1.35, 1.21

a The number of lognormal modes fitted to each distribution is listed in the first column; subsequent columns report the number concentration (*N_i_*), geometric mean diameter (*D*_g_), and geometric standard deviation (σ_g_) for each mode. Lognormal fittings are depicted in Figure S8.

In contrast, rBC size distributions from the APP data sets are more complex with multiple modes identified in each case (Table 2). The APP campaign average and morning rush hour distributions exhibit visual similarity (Figure S8) and share a dominant mode diameter (i.e., the diameter corresponding to the most prominent mode in each distribution) around 90 nm (Table 2), suggesting that traffic emissions also contribute substantially to rBC concentrations in the rural environment. In addition to traffic-related emissions, rBC concentrations at the APP site appear to be influenced by biomass burning sources, as the dominant mode diameters for APP rBC peak 1 and peak 2 align with larger particle sizes reported in previous studies for biomass burning rBC mass distributions.^64^ These peaks are therefore interpreted as biomass burning episodes, potentially associated with localized events given their relatively short durations. No other major biomass burning events were observed during the study period.

Total rBC and FeO*_x_* population *b*_abs_ derived from SP2 observations and corresponding lognormal fits are presented in Table 1. Because the fits extend beyond the SP2′s measured size range, fitting results consistently yield higher total *b*_abs_ than the direct SP2 measurements for both species. Absorption is substantially greater at the ATL site compared to the rural APP site, particularly for FeO_*x*_, where the fitted *b*_abs_ is more than 5 times higher. At the ATL site, the relative absorption by FeO_*x*_ compared to rBC remains approximately 1.3% for both measured and lognormally fitted size distributions. At the APP site, this ratio increases from 0.5 to 0.6% due to the extended size range from lognormal fitting.

Furthermore, both rBC and FeO_*x*_ size distributions are shifted toward larger sizes at the ATL site compared to at the APP site (Figure 5). The prevalence of smaller rBC and FeO_*x*_ particles at APP suggests the influence of long-range transport on pollutant levels at this site, as larger particles are removed more efficiently during transport.^65,66^ Biomass burning events, which often release fine iron-containing particles,^67,68^ may also explain the smaller FeO*_x_* particles observed at APP. However, while the SP2 effectively captures the bulk of the rBC population (Figure 5), a substantial fraction of the FeO_*x*_ population lies outside of the instrument’s detectable range and contributes significantly to total FeO_*x*_ mass and absorption. Lognormal fitting results increase FeO_*x*_ mass by up to a factor of 2 (Table 1), indicating that SP2-derived mass estimates represent lower limits.

**Figure 5:**
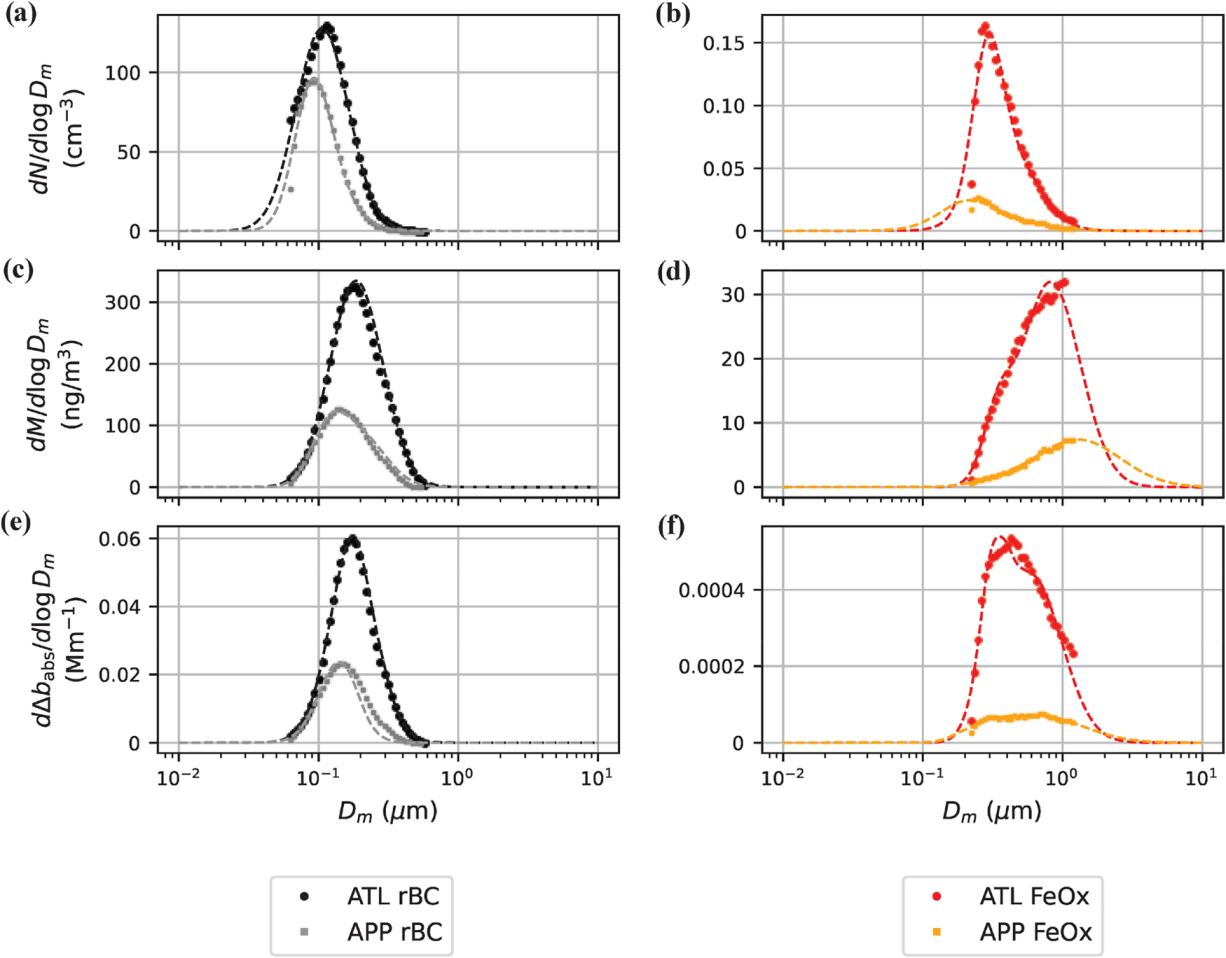
Average rBC and FeO_*x*_ size distributions and absorption as functions of particle diameter for Atlanta, GA and Boone, NC sites. (a) rBC number distribution, (b) FeO_*x*_ number distribution, (c) rBC mass distribution, (d) FeO_*x*_ mass distribution, (e) rBC absorption as a function of diameter, (f) FeO_*x*_ absorption as a function of diameter. Measured data are shown as scatter points, and corresponding lognormal fits are shown as dashed lines.

### 3.4 Uncertainty Estimates

Based on the various calibration results (Table S1), the uncertainty in reported average rBC mass is ±1.2%, while the uncertainty for average FeO*_x_* mass is significantly larger (±46%) due to calibration-related instrument variability. Additionally, assuming all FeO_*x*_ particles are Fe_3_O_4_ (magnetite) rather than Fe_2_O_3_ (hematite) introduces an uncertainty of ±13% in the reported average FeO_*x*_ mass concentration resulting from differences in particle density and calibration factors. Despite this uncertainty in FeO_*x*_ mass, however, the assumption that all FeO_*x*_ particles are Fe_2_O_3_ rather than Fe_3_O_4_ results in only a 0.1% decrease in the relative absorption by FeO_*x*_ compared to rBC due to the larger scattering and smaller absorbing components of the refractive index for Fe_2_O_3_ compared to Fe_3_O_4_.

## 4 Discussion

In this study, we examine the temporal variability, correlations, size distributions, and relative absorption of rBC and FeO_*x*_ concentrations at two contrasting sites in the southeastern U.S.: a high-elevation rural environment (APP) and an urban environment (ATL). Our findings highlight notable differences in pollutant concentrations and their relationship with wind speed and direction between these settings. rBC and FeO_*x*_ concentrations and absorption are substantially higher at the urban site, reflecting significant anthropogenic contributions to pollutant emissions. At the rural site, the predominance of smaller particle sizes suggests the influence of long-range transport; however, the distinct weekday–weekend pattern in the diurnal rBC concentration profile indicates that local anthropogenic sources also contribute significantly to pollutant levels.

Given the SP2′s low sensitivity to natural mineral dust with mixed morphology and composition^2,33,34^ and its detection of iron-containing particles primarily within the submicrometer size range, the FeO_*x*_ particles observed in this study are most consistent with combustion-related and other anthropogenic sources (e.g., traffic emissions, industrial activity, and biomass burning), rather than coarse-mode mineral dust. At the urban ATL site, the clear weekday–weekend diurnal pattern and coemission with rBC suggest that traffic-related anthropogenic emissions are a dominant source of FeO_*x*_. In contrast, at the rural high-elevation APP site, the smaller particle sizes observed and the lack of a clear relationship between pollutant concentrations and wind speed suggest that long-range transport is a more important contributor. Although some source information can be indirectly inferred from temporal variability and particle size distributions at both sites, identifying exact FeO_*x*_ sources remains challenging based on these observations alone.

The observed size distributions of rBC and FeO_*x*_ in this study are generally consistent with prior measurements in other regions. Aircraft-based measurements over East Asia report rBC number and mass modes near ∼100 nm and 150–200 nm,^38^ respectively, as well as FeO_*x*_ number modes of ∼100–300 nm and mass distributions extending into the supermicrometer range.^2^ These modal ranges are consistent with those observed in this study. While our measurements are constrained by the SP2 detection range, the truncated FeO_*x*_ distributions suggest that a substantial fraction of FeO_*x*_ mass lies outside the detectable size range, consistent with previous observations.^2^

By extending the SP2 size range using lognormal fitting, we find that FeO_*x*_ contributes approximately 1.3% of rBC absorption at ATL and 0.6% at APP, suggesting that anthropogenic FeO_*x*_ contributes about twice as much to total shortwave absorption at the urban site compared to the rural site. These values are lower than the range of estimates reported by Moteki et al., who found that absorption by anthropogenic FeO_*x*_ contributes 4–7% of the absorption attributed to rBC over urban Southeast Asia.^2^ This discrepancy is consistent with differences in regional anthropogenic activity, as population density and associated anthropogenic emissions are substantially higher in Southeast Asia compared to the southeastern U.S.^69–71^ Despite relatively lower absolute concentrations in ATL compared to more polluted regions, the Atlanta area remains a regional hotspot for rBC in the continental U.S.^7,45^ These levels are linked to significant health^7^ and radiative impacts,^72^ underscoring the importance of understanding the covariability of rBC with species such as FeO_*x*_ that are similarly associated with adverse health outcomes^4,5^ and non-negligible contributions to shortwave absorption.^2,27^ Another study by Lamb et al. reports that atmospheric heating by anthropogenic FeO_*x*_ ranges from 0.3 to 26% of that of rBC in remote environments,^34^ which is supported by our findings in the rural setting.

Several limitations of this study should be considered. First, uncertainties arise from different instrument calibrations due to changes in instrument sensitivities. Our sensitivity analysis using calibrations before and after the APP campaign suggests a ±1.2% uncertainty for rBC mass and a ±46% uncertainty for FeO_*x*_ mass due to calibration differences. Although the change in instrument sensitivity may affect the comparability of rBC and FeO_*x*_ mass measurements, the measured number concentrations should not be affected, as they are not dependent on the calibration. Additionally, the lack of specificity regarding the types of ambient FeO_*x*_ measured introduces further uncertainty in estimated FeO_*x*_ mass (±13%), as we cannot determine which specific iron oxides are present in the atmosphere. However, our sensitivity analysis using magnetite and hematite indicates lower uncertainty in estimated light absorption. Under the assumption that all FeO_*x*_ particles are hematite rather than magnetite, the measured FeO_*x*_ absorption relative to rBC decreases to 0.4%, reflecting hematite’s lower absorption index. Despite these uncertainties, the observed spatial and temporal patterns in rBC and FeO_*x*_ remain consistent across all sensitivity cases.

In addition, our study primarily measures iron within the PM_1_ fraction, excluding larger particles. Within this size range, the SP2 may only be sensitive to highly absorptive particles with a high Fe fraction, which generate strong incandescence signals measurable by the instrument. This measurement approach may therefore preferentially detect anthropogenic Fe particles with nearly pure FeO_*x*_ while being less responsive to natural mineral dust with relatively low Fe content. As a result, the FeO_*x*_ mass concentration detected by SP2 represents only a small fraction of total Fe-containing particles. Recent studies^73,74^ have reported a summer average total Fe concentration of approximately 90 ng/m^3^ in Atlanta, compared to the FeO_*x*_ mass concentration of 15 ng/m^3^ measured in this study, highlighting the difference between our measurements and those of total Fe concentrations.

Moreover, our assumption of an external mixing state for absorption calculations may not fully represent the actual mixing state of particles in the atmosphere given the complexity and variability of particle size, mixing state, and composition. While information on the mixing state of FeO_*x*_ remains limited, internal mixing of rBC with nonrefractory coatings is known to enhance rBC light absorption by up to a factor of ∼2 through lensing effects.^75–80^ Thus, the assumption of an external mixing state provides a useful first-order estimate for calculating absorption, but may introduce uncertainty in the relative contributions of rBC and FeO_*x*_. Consequently, our absorption estimates likely represent lower bounds under internally mixed conditions. These assumptions should be considered when interpreting the results and underscore the need for further studies to refine the measurements and assumptions used in this analysis.

When comparing BC concentrations measured by the SP2 and aethalometer at the APP site (Figure S6), SP2 values are approximately 50% lower than those measured by the aethalometer. This discrepancy is consistent with findings from previous studies^81–83^ and likely reflects fundamental differences in measurement principles. The SP2 detects individual rBC particles based on laser-induced incandescence and is optimized for mass-resolved detection, but it may undersample particles with small core diameters or those outside the calibrated size range. In contrast, the aethalometer indirectly estimates BC mass by measuring light absorption by all aerosol components collected on a filter and applying a mass absorption cross-section. This approach may include contributions from nonrefractory BC light-absorbing particles such as dark brown carbon or internally mixed species, potentially leading to overestimation of BC concentrations. These factors suggest that, while the SP2 offers high sensitivity for detecting rBC, its measurements may represent a lower bound of total light-absorbing carbon, especially in complex ambient aerosol environments.

Despite the noted limitations, observations from the two study sites highlight significant differences between the high-elevation rural and urban environments, illustrating the influence of both local and regional factors on pollutant levels. While anthropogenic emissions are important at both sites, their effects are shaped by different factors: concentrated local emissions in the urban ATL environment, and contributions from both local and long-range sources in the rural APP setting. These findings contribute to our understanding of air quality dynamics across different environments and underscore the need for further research to improve measurement accuracy and address uncertainties.

## Supplementary Material



## Notes

Tabulated statistics and uncertainty estimates for rBC and FeO*_x_* measurements; figures of site locations and details; additional pollutant and meteorological timeseries data; SP2 calibration curves; particle classification methodology; relationships between pollutant concentrations and wind conditions; SP2–aethalometer comparisons; and normalized rBC size distributions with corresponding lognormal fits (PDF)
