# Supplementary material for: Assessing Black Carbon and Iron Oxide Aerosols: A Comparative Study between Urban and Rural Environments in the Southeastern U.S
Source: ACS EST Air. 2026 May 22;3(6):1485–96. doi: 10.1021/acsestair.5c00400 (PMC13270512; doi:10.1021/acsestair.5c00400)
Supplement: Supplementary file 1 [file ea5c00400_si_001.pdf]

Supporting Information for:

Assessing Black Carbon and Iron Oxide Aerosols: A Comparative Study Between Urban and Rural Environments in the Southeastern U.S.

by

Shreya Suri (1), Lifei Yin (1), Bin Bai (1), Yuhan Yang (1), Dongli Wang (2), Andrew R. Metcalf (2), Nathan Chellman (3), Robert F. Swarthout (4), James Sherman (5), and Pengfei Liu* (1)

(1) School of Earth and Atmospheric Sciences, Georgia Institute of Technology, Atlanta, GA 30332, USA
(2) Department of Environmental Engineering and Earth Sciences, Clemson University, Clemson, South Carolina, USA
(3) Division of Hydrologic Sciences, Desert Research Institute, Reno, NV 89512, USA
(4) Department of Chemistry and Fermentation Sciences, Appalachian State University, Boone, NC 28608, USA
(5) Department of Physics and Astronomy, Appalachian State University, Boone, NC 28608, USA

E-mail: pengfei.liu@eas.gatech.edu

Environmental Science and Technology

*To Whom Correspondence Should be Addressed

Supplementary Tables and Figures

	Pre-campaign rBC	Post-campaign rBC	Pre-campaign FeOx (assumed as Fe_3O_4)	Post-campaign FeOx (assumed as Fe_3O_4)	Post-campaign FeOx (assumed as Fe_2O_3)
Average mass (ng/m^3)	59.0	58.3	1.92	3.53	3.06
Uncertainty	0.012		0.456	0.133	

Table S1. Summary of average rBC and FeOx mass concentrations and associated uncertainties during the APP campaign. "Pre-campaign" and "Post-campaign" refer to values determined using pre- and post-campaign calibrations, respectively (see **Figure S3** for calibration curves). For FeOx, post-campaign average mass concentrations are reported assuming either all particles are magnetite (Fe_3O_4) or all particles are hematite (Fe_2O_3). Uncertainty values are calculated as $\Delta x/x$, where Δx is the difference of the two values indicated by the braces.

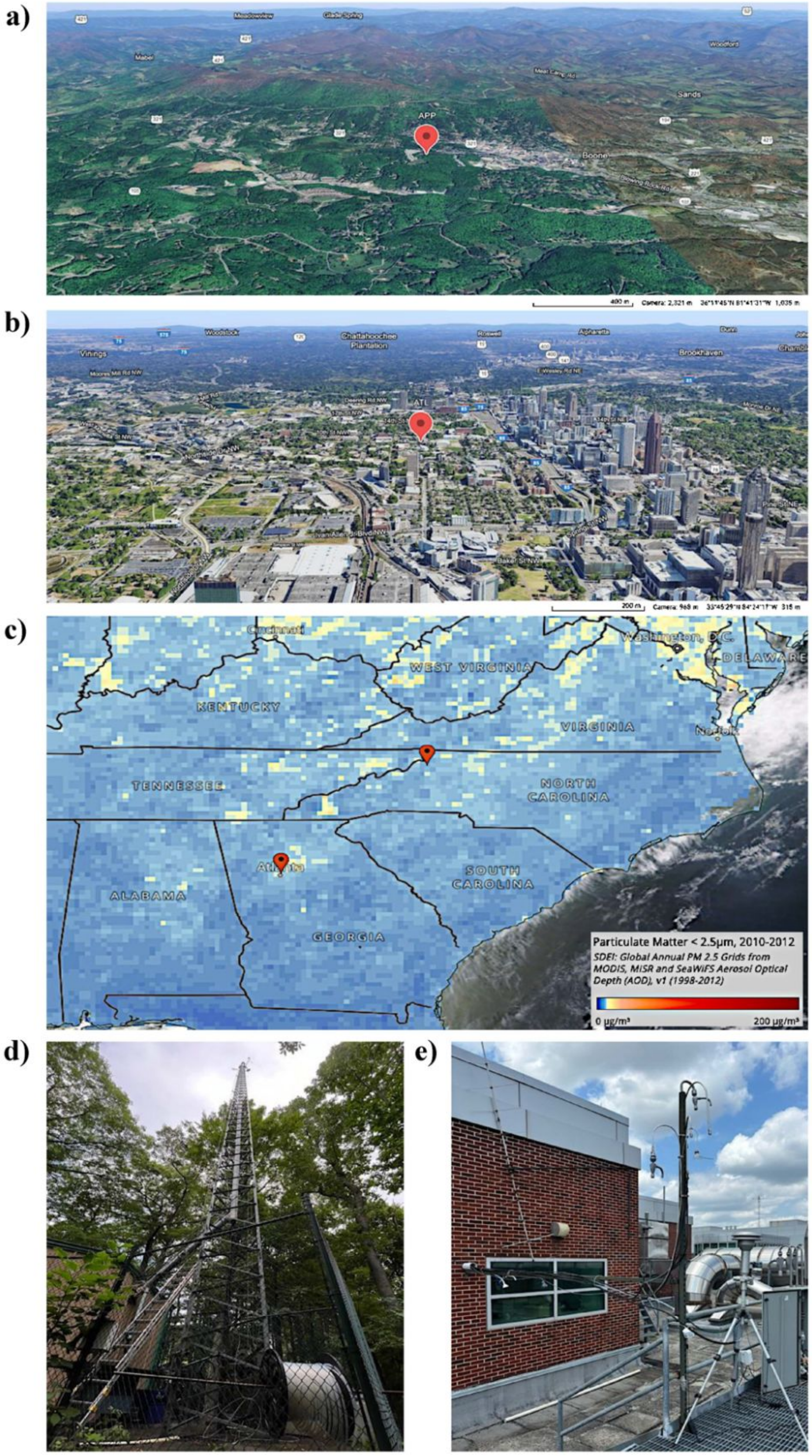

Figure S1. Study locations and monitoring sites. **a, b)** 3D satellite views of APP[1] and ATL[2] sites obtained from Google Earth. **c)** Map of the southeastern U.S showing geographic locations of Boone, NC and Atlanta, GA along with global annual $PM_{2.5}$ averages obtained from the NASA

Worldview application[3]. **d)** 30-meter tower at the AppalAIR facility in Boone, NC, used for continuous atmospheric monitoring. **e)** Ambient monitoring station on the rooftop of the Environmental Science and Technology building on the Georgia Tech campus in Atlanta, GA.

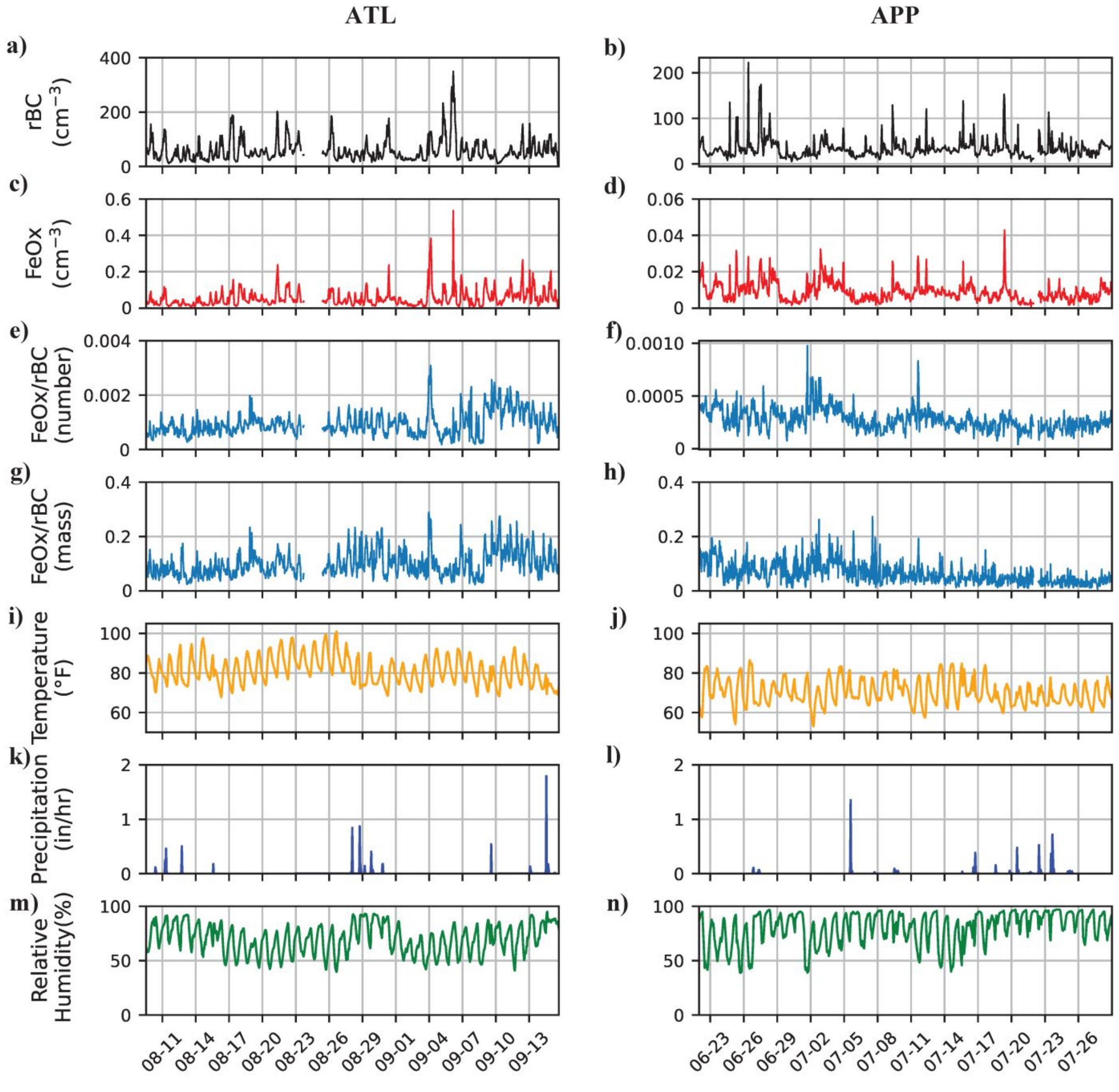

Figure S2. Timeseries of rBC and FeOx concentration and meteorological variables at Atlanta, GA and Boone, NC sites. **a, b)** rBC number concentration, **c, d)** FeOx number concentration, **e, f)** FeOx/rBC number concentration ratio, **g, h)** FeOx/rBC mass concentration ratio, **i, j)** temperature, **k, l)** precipitation rate, and **m, n)** relative humidity at Atlanta and Boone sites, respectively. Meteorological data were obtained from Georgia Tech Bobby Dodd Stadium and Appalachian State University Kidd Brewer Stadium WeatherSTEM sites[4,5].

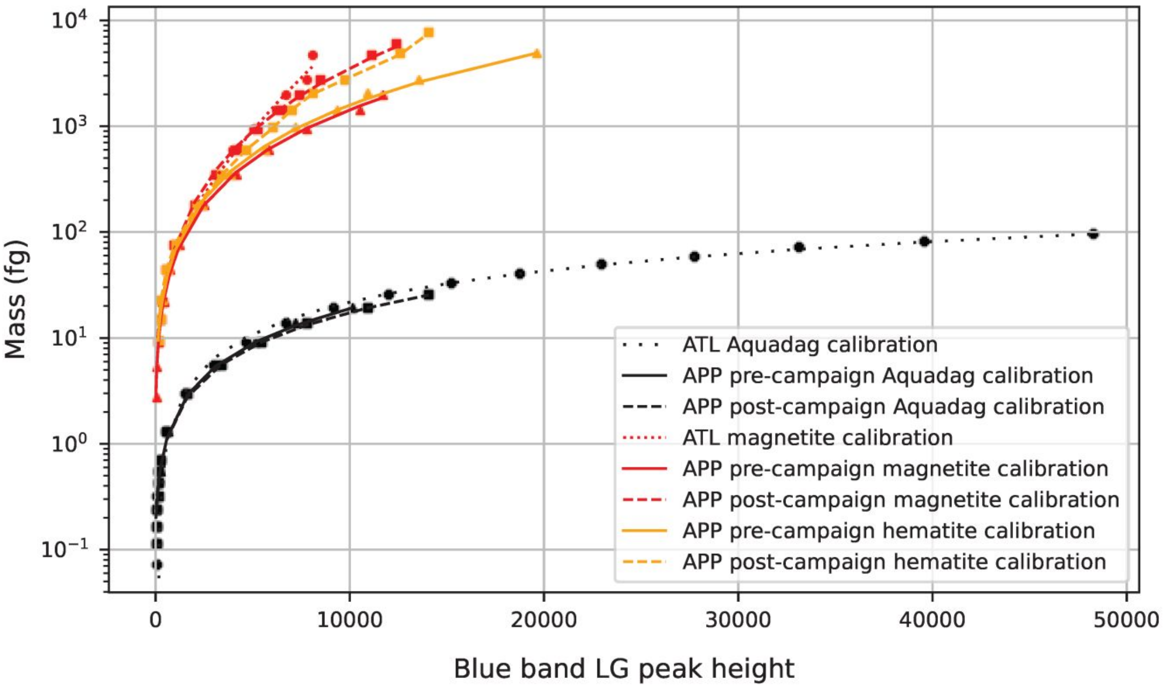

Figure S3. SP2 calibration curves for Aquadag (black lines), magnetite (red lines), and hematite (orange lines) with calculated particle mass plotted against the blue band low gain (LG) signal. ATL Aquadag, ATL magnetite, and APP post-campaign Aquadag and magnetite calibrations were used for the main analysis, while APP pre-campaign calibrations and post-campaign hematite calibrations were used for uncertainty analysis.

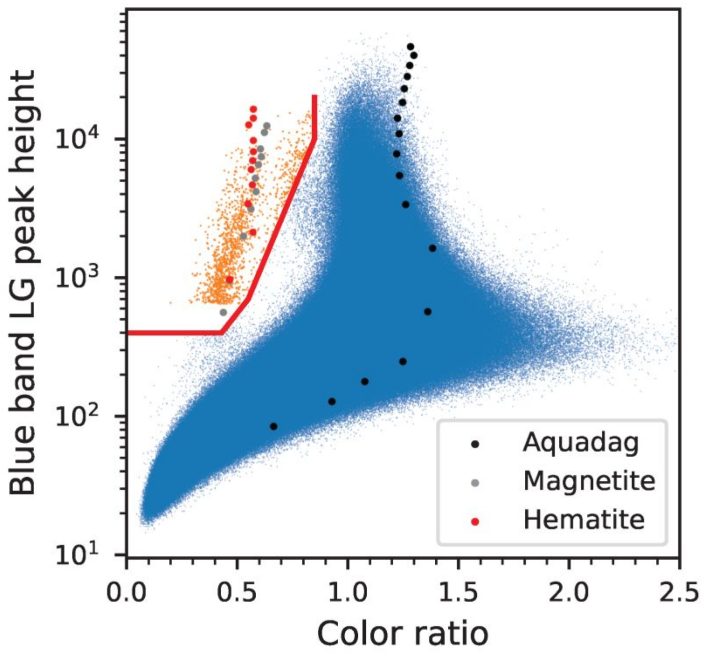

Figure S4. Scatter plot of color ratios for rBC and FeOx particles measured on July 22, 2024 during the APP campaign. The color ratio represents the SP2 blue band/red band signal ratio. The red boundary line distinguishes FeOx particles (orange) from rBC particles (blue). Post-campaign Aquadag (black marker), magnetite (gray marker), and hematite (red marker) calibration data are also plotted. This classification method follows the methodology of Moteki et al. (2017).[6]

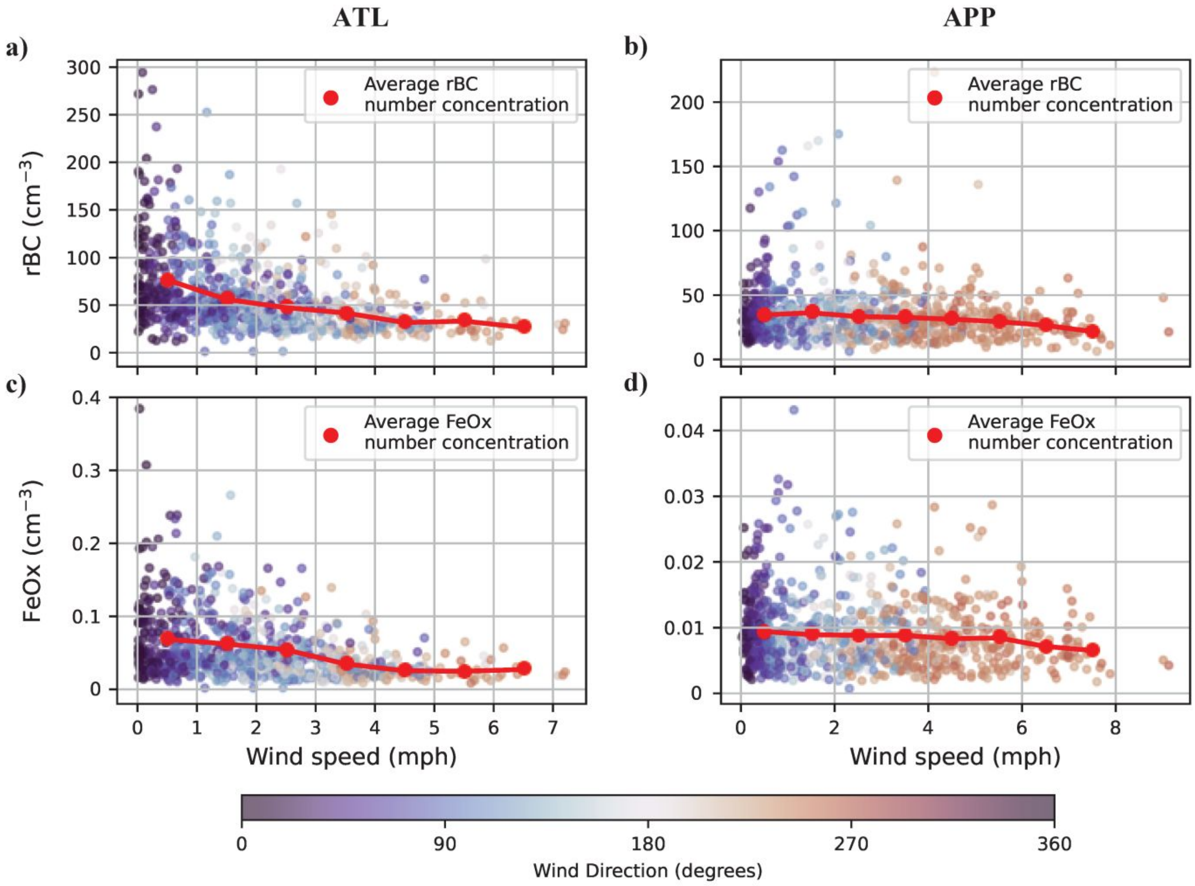

Figure S5. Relationship between pollutant number concentration, wind speed, and wind direction. Scatterplots of **a, b)** rBC and **c, d)** FeOx number concentration plotted against wind speed for ATL and APP sites. Colored markers represent wind direction, while larger red markers represent mean pollutant number concentration for each bin. Wind data were obtained from Georgia Tech Bobby Dodd Stadium and Appalachian State University Kidd Brewer Stadium WeatherSTEM sites[4,5].

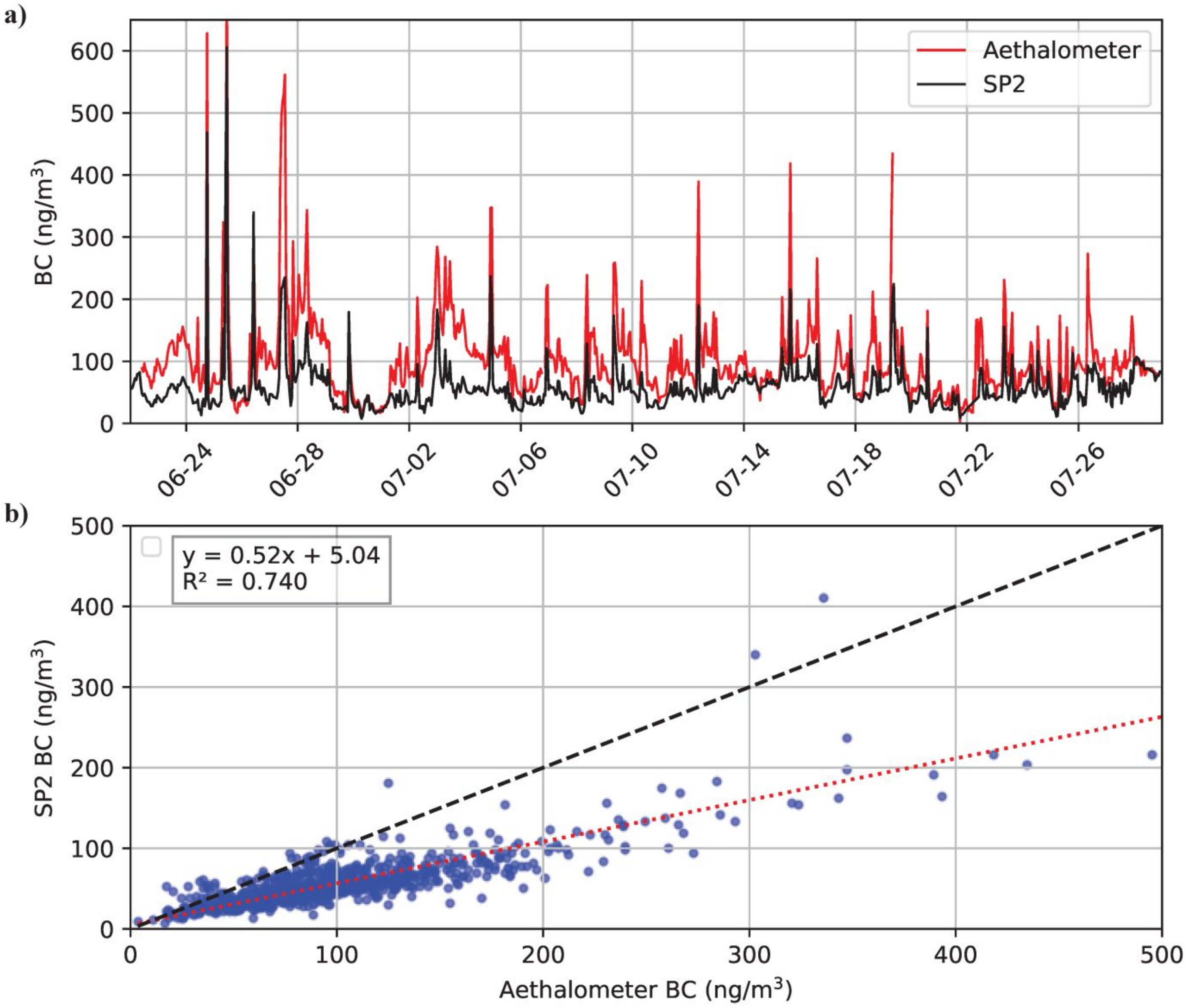

Figure S6. Comparison of BC mass concentration measurements by SP2 and aethalometer (λ = 880 nm) at APP site. **a)** Timeseries of BC mass concentration measured by the aethalometer at 880 nm (red line) and SP2 (black line). **b)** Correlation between aethalometer and SP2 BC mass concentration measurements with trendline (red line) and identity line (black line) shown.

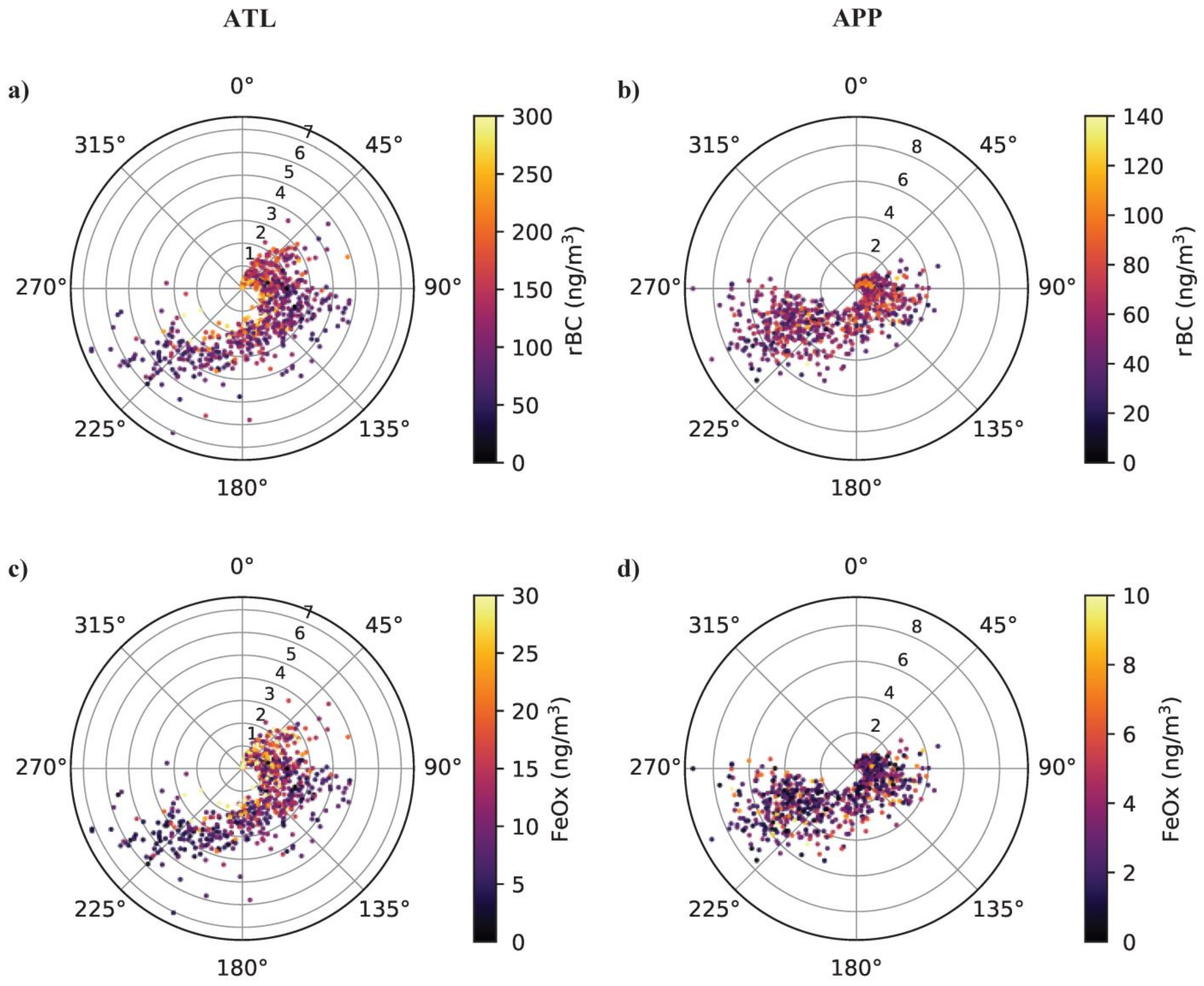

Figure S7. Relationship between pollutant mass concentration, wind speed, and wind direction. Pollutant roses show hourly **a, b)** rBC and **c, d)** FeOx mass concentration measurements for ATL and APP sites. Concentric rings represent wind speed intervals (in mph). Wind data were obtained from Georgia Tech Bobby Dodd Stadium and Appalachian State University Kidd Brewer Stadium WeatherSTEM sites[4,5].

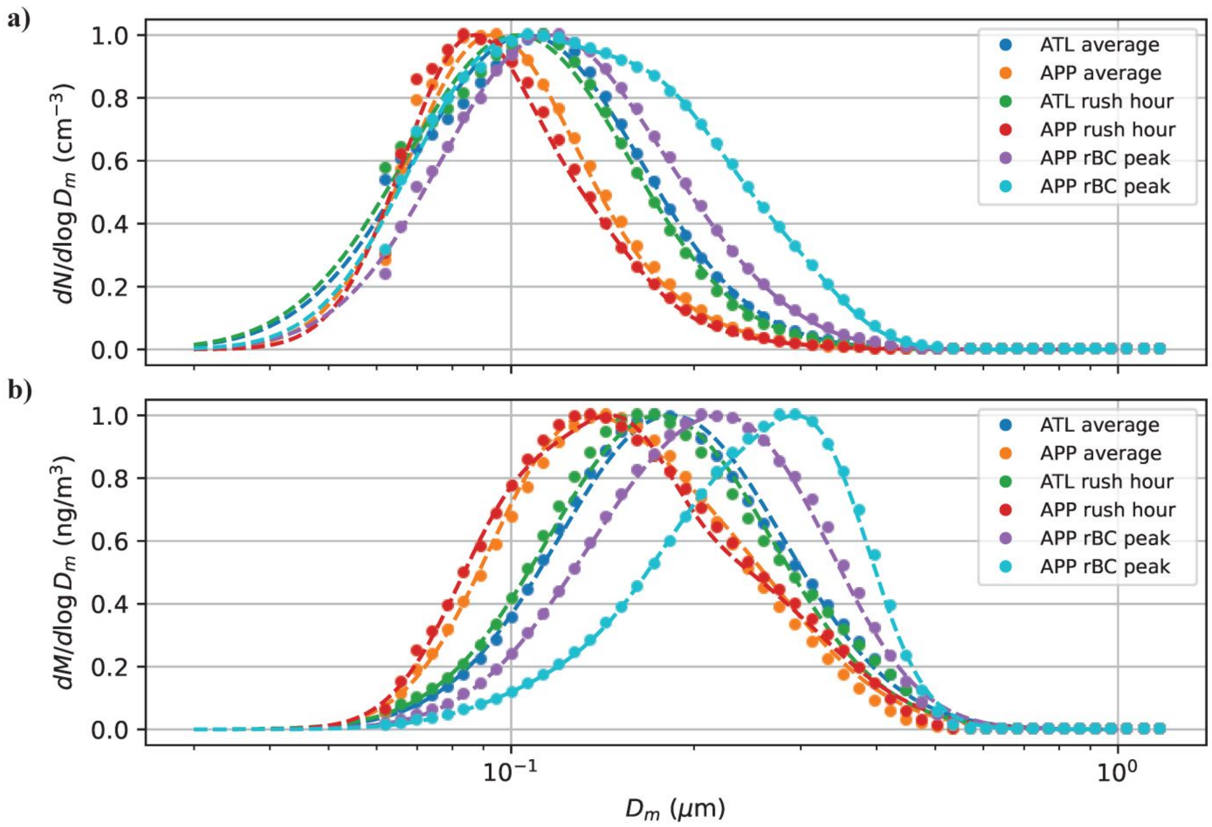

Figure S8. Normalized rBC number and mass distributions for multiple datasets, including ATL campaign average, APP campaign average, ATL morning rush hour, APP morning rush hour, APP rBC peak 1, and APP rBC peak 2. Measured data are shown as scatter points, and corresponding lognormal fits are shown as dashed lines.

Supplementary References

(1) Google Earth. Satellite imagery of AppalAIR in Boone, NC. http://earth.google.com (accessed 2024-08-18).

(2) Google Earth. Satellite imagery of the Ford Environmental Science and Technology building in Atlanta, GA. http://earth.google.com (accessed 2024-08-18).

(3) NASA Worldview. SDEI: Global Annual PM2.5 Grids from MODIS, MISR, and SeaWiFS Aerosol Optical Depth (AOD), v1 (1998–2012). https://worldview.earthdata.nasa.gov/ (accessed 2025-09-16).

(4) WeatherSTEM. Georgia Tech Bobby Dodd Stadium. https://gatech.weatherstem.com/data (accessed 2024-08-16).

(5) WeatherSTEM. Appalachian State University. https://watauga.weatherstem.com/data (accessed 2024-08-16).

(6) Moteki, N.; Adachi, K.; Ohata, S.; Yoshida, A.; Harigaya, T.; Koike, M.; Kondo, Y. Anthropogenic Iron Oxide Aerosols Enhance Atmospheric Heating. *Nat. Commun.* **2017**, *8* (1), 15329. https://doi.org/10.1038/ncomms15329.
